# Phenylphenalenones Accumulate in Plant Tissues of Two Banana Cultivars in Response to Herbivory by the Banana Weevil and Banana Stem Weevil

**DOI:** 10.3390/plants5030034

**Published:** 2016-08-25

**Authors:** Dirk Hölscher, Andreas Buerkert, Bernd Schneider

**Affiliations:** 1 Organic Plant Production and Agroecosystems in the Tropics and Subtropics, University of Kassel, Steinstr. 19, 37213 Witzenhausen, Germany; buerkert@uni-kassel.de; 2 Max-Planck Institute for Chemical Ecology, Hans-Knöll-Str. 8, 07745 Jena, Germany; schneider@ice.mpg.de

**Keywords:** *Cosmopolites sordidus*, *Odoiporus longicollis*, Musaceae, phenylphenalenones, 2-methoxy-4-phenylphenalen-1-one

## Abstract

Phenylphenalenone-type compounds accumulated in the tissues of two banana cultivars—*Musa acuminata* cv. “Grande Naine” (AAA) and *Musa acuminata* × *balbisiana* Colla cv. “Bluggoe” (ABB)—when these were fed on by the banana weevil (*Cosmopolites sordidus* (Germ.) (Coleoptera: Curculionidae)) and the banana stem weevil (*Odoiporus longicollis* (Oliver) (Coleoptera: Curculionidae)). The chemical constituents of the banana material were separated by means of chromatographic techniques and identified by NMR spectroscopy. One new compound, 2-methoxy-4-phenylphenalen-1-one, was found exclusively in the corm material of “Bluggoe” that had been fed on by the weevils.

## 1. Introduction

**Figure 1:**
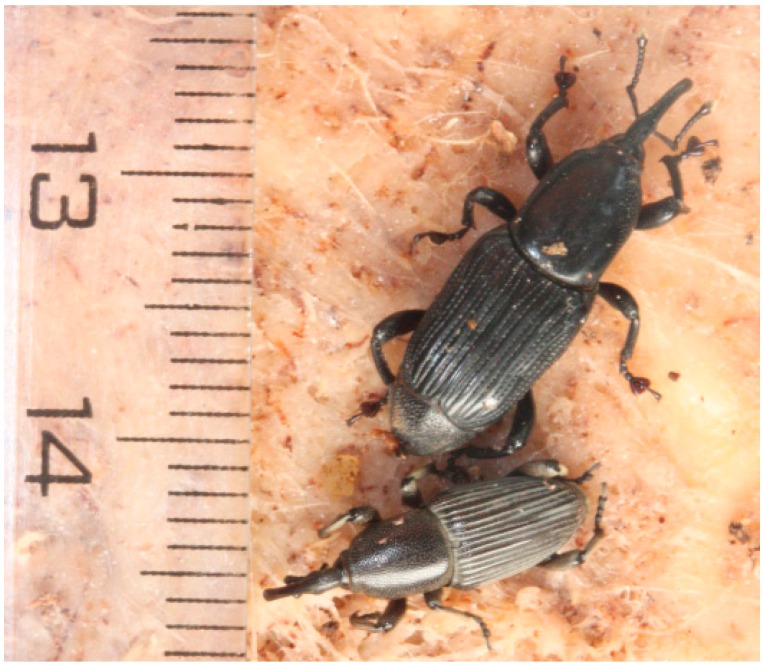
Adult banana (pseudo)stem weevil, *Odoiporus longicollis* (OL)—larger weevil facing top right corner of photo and adult banana weevil, *Cosmopolites sordidus* (CS)—smaller weevil at bottom of photo facing ruler.

Bananas (*Musa* spp.) are a major staple food for more than 400 million people in developing countries in the tropics and subtropics, and an important export product for the Philippines and many Latin American countries [1,2]. Production is threatened by a variety of pests and pathogens, such as plant parasitic nematodes, ascomycetous fungi, viruses, bacteria, and insects [3,4,5,6,7,8]. The most important banana production and productivity limiting insect-pests are the banana weevil, *Cosmopolites sordidus* (CS), and the banana (pseudo)stem weevil, *Odoiporus longicollis* (OL) [9]. Adult weevils can be distinguished from one another by differences in body length, body width, and rostrum length: CS has a shorter and less slender rostrum, and OL a broader pronotum tapered anterior and a broader third tarsal segment (Figure 1) [10]. CS is native to Southeast Asia, but has been transported throughout the banana growing regions of the world, and is now established in southern Asia, Africa, many Pacific islands, Australia, northern South America, most of Central America, Cuba, the West Indies, and in Mexico, Florida, and Hawaii [11]. OL is also native to Southeast Asia, but its distribution is restricted to the banana growing belts of India, Southeast Asia, southern China, and the southern islands of Japan.

CS is regarded as the main insect pest in bananas [11], though OL is a major limiting factor of banana production in India, which is the country that produces the most bananas worldwide. OL causes 10%–90% yield losses, depending upon the growth stage of the crop and management efficiency [12,13]. CS larvae create complex tunnel systems in the corm and rhizome of banana plants, leading to their toppling, often shortly before fruit harvest. In contrast, the larvae of OL produce long tunnels in the banana pseudostem, weakening the plant, reducing flowering rates, and finally producing undersized or no fruit [12]. Recently, phenylphenalenones have been identified as effective plant secondary metabolites involved in the defense response of particular banana cultivars to both the burrowing nematode (*Radopholus similis*) [14] and the fungal disease Black Sigatoka, caused by *Mycosphaerella fijiensis* [15]. The biosynthesis of the phenylphenalenones is closely related to that of flavonoids. Both classes of natural products are derived from the phenylpropanoid/plant polyketide pathway [16,17]. The present study focuses on phenylphenalenone-type compounds produced by two different banana varieties, *Musa acuminata* cv. “Grande Naine” (GN) and *M. acuminata* × *balbisiana* Colla cv. “Bluggoe” (BG) in reaction to banana weevils (CS) and banana pseudostem weevils (OL) feeding on the plants.

## 2. Results

**Figure 2:**
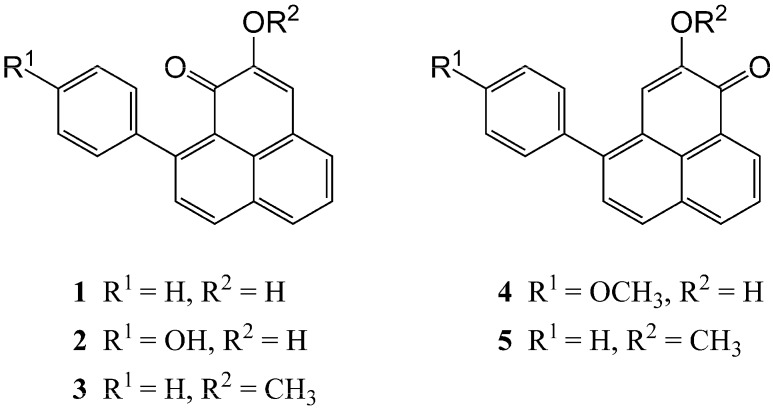
Structures of phenylphenalenone-type phytoalexins (**1**–**5**). (**1**) Anigorufone; (**2**) hydroxyanigorufone; (**3**) methoxyanigorufone; (**4**) 4-*O*-methylirenolone; (**5**) 2-methoxy-4-phenylphenalen-1-one isolated from pseudostem or corm material from *Musa* AAA cv. “Grande Naine” and *Musa* ABB cv. “Bluggoe” that had been fed on by *Odoiporus longicollis* or *Cosmopolites sordidus*.

Six weeks after starting the feeding experiments, the banana pseudostem and corm material of BG and GN were inspected for damage caused by the beetles. A change of the coloration from green to orange-red in the case of the pseudostem-material and from pale-brown to dark red-brown for the corm material was detected. Control material of BG and GN showed no such discoloration. Secondary metabolites were extracted from the plant material using ethanol. Liquid–liquid separation of all the samples and controls resulted in CHCl_3_, ethyl acetate, 1-butanol, and aqueous subfractions. Similar to the procedure of nematode-infected banana material [14], only the CHCl_3_ subfractions of the wounded plant material contained phenylphenalenones. These CHCl_3_ subfractions were separated by thin-layer chromatography. The purified compounds were subjected to ^1^H NMR spectroscopy. The structures of all the compounds (Figure 2) were identified as phenylphenalenones, which are typical metabolites and major phytoalexins of *Musa* species. Compounds (**1**–**4**) (Figure 2) have been previously reported [18], and 2-methoxy-4-phenylphenalen-1-one (**5**) is a new natural product.

**Figure 3:**
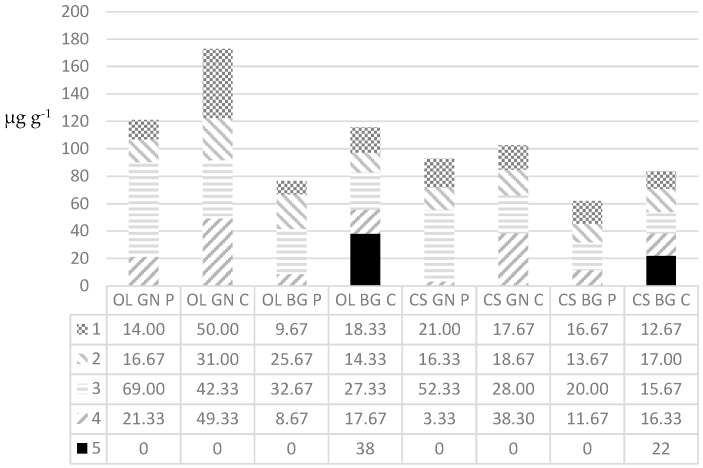
Average masses (µg) of the five phenylphenalenones (**1**–**5**) detected per g pseudostem (P) or corm material (C) from *Musa* AAA cv. “Grande Naine” (GN) or *Musa* ABB cv. “Bluggoe” (BG) after being fed on by either *Cosmopolites sordidus* (CS) or *Odoiporus longicollis* (OL) for 6 weeks. The values are the averages of two very similar measured masses.

Generally, the corm material contained greater quantities of secondary metabolites than pseudostem material (Figure 3).

Generally, for each weevil and cultivar combination, greater quantities of phenylphenalenones were produced in corm material than in pseudostem material, with GN corm material fed on by OL producing the greatest cumulative mass and with BG material fed on by CS producing the smallest cumulative mass. The corm material of GN always produced greater cumulative quantities than the BG corm pieces for both the weevil species. Compounds **1**, **2**, **3**, and **4** occur in greater quantities in GN- than BG corm material fed on by OL or CS. The same results were observed with pseudostem material, with the exception of **2** and **4** for BG pseudostem material. The new compound **5** was only detectable in relatively high quantities in the BG-corm material, fed on by CS or OL. The structural elucidation of **5** followed that for 2-hydroxy-4-phenylphenalen-1-one reported by [19]. The position of the additional 2-*O*-methyl group of **5** was confirmed by the Heteronuclear Multiple-Bond Correlation (HMBC) cross signal between the methoxy protons (δ 3.72) and C-2 (δ 153.8).

## 3. Discussion

Phenylphenalenones have been identified as typical phytoalexins of *Musa* species [18]. The objective of this study was to investigate which defense metabolites are produced by two *Musa* cultivars (GN and BG) in response to herbivory by two important banana insect pests (OL and CS). The phytochemical profiles of the different combinations of plant material, cultivar, and weevil revealed that each treatment produced a unique mix of **1**–**5**, with none of these metabolites dominating across treatments. The concentration of a specific compound can be crucial in the effectiveness of the defense response of plant materials attacked by pests and pathogens, as was shown for **1** in the interaction of the burrowing nematode *R. similis* with two different *Musa* cultivars with different levels of susceptibility [14]. Recently, the role of methoxyanigorufone (**3**) and the non-methylated analogue of **5**, isoanigorufone, were reported to be the most relevant metabolites for the defense of *Musa* plants against *M. fijiensis* [15]. The new phenylphenalenone, 2-methoxy-4-phenylphenalen-1-one (**5**) was only produced in BG-corm material, and may also play a role in the defense of *Musa* spp. against weevil attack. However, further studies are needed to confirm the importance of this compound in the plant–pest interaction.

## 4. Materials and Methods

### 4.1. Plant Material

“Grande Naine” (ITC1256, GN) in vitro plantlets were provided by the International Transit Centre (ITC), Bioversity International, Katholieke Universiteit Leuven, Belgium. Plants with roots were first transplanted into small pots containing a sterile quartz–peat mixture (2:1). At weekly intervals, each plant received a dose of commercial liquid fertilizer mixture of 2% Ferty^®^ 3 (Planta Düngemittel GmbH, Regenstauf, Germany; www.plantafert.de) and 0.2% Folicin-Bor (Jost GmbH, Iserlohn, Germany; www.jost-group.com). Additionally, an aqueous solution of 0.05% Biplantol^®^ agrar (Bioplant Naturverfahren GmbH, Konstanz, Germany; www.biplantol.com) was applied. Later, the plants were transplanted into larger 8 L pots containing the same potting mix and were maintained under greenhouse conditions at an ambient temperature of 27/20 °C (day/night), 80% relative humidity, 12-h photoperiod, and irrigated as needed. Suckers of “Bluggoe” (BG) plants were originally collected in Oman [20] and cultivated under the same greenhouse conditions as described for GN.

### 4.2. Banana Weevils

Adult CS were originally provided from the International Institute of Tropical Agriculture (IITA), Kampala, Uganda. Adult OL were provided by the Department of Agronomy, Yezin Agriculture University, Yezin, Nay Pyi Taw, Myanmar. The weevils were reared and propagated in small plastic boxes (inside diameter 11 cm) on wet tissue paper and fed with small pieces of pseudostem material from different *Musa* cultivars. These plastic boxes were kept in a growth chamber at an ambient temperature of 26 °C, 55% relative humidity, and a 16-h photoperiod.

### 4.3. Weevil Feeding Experiments

A total of 16 feeding containers were used, each containing 30 g of either pseudostem or corm material from either GN or BG and four adult CS or OL. Additionally, the same number of containers (16) without weevils served as non-feeding control. Each week, the containers were carefully sprayed with water. Six weeks after set-up, the banana material pieces left in the 32 containers were snap frozen in liquid nitrogen and stored at −80 °C in preparation for phytochemical analysis.

### 4.4. Isolation and Structure Elucidation of Phenylphenalenones

The frozen banana material was ground and exhaustively extracted with ethanol at room temperature. The crude extract was evaporated (<40 °C) and partitioned between CHCl_3_-H_2_O and ethyl-acetate-H_2_O, followed by 1-butanol-H_2_O. Purifications were achieved by means of thin layer chromatography (TLC: silica gel 60 F_254_, toluene–Me_2_CO (2:1)).

### 4.5. NMR Spectroscopy

The individually collected compounds were subjected to ^1^H NMR spectroscopy for identification. All compounds were identified as phenylphenalenones (**1**–**5**, Figure 2). Metabolites **1**–**4** are known typical metabolites and major phytoalexins of *Musa* species, while 2-methoxy-4-phenylphenalen-1-one (**5**) is reported here for the first time. ^1^H NMR and 2D NMR spectra (^1^H-^1^H COSY, HSQC, HMBC) were measured on an AV 500 NMR spectrometer (Bruker, www.bruker.com) at 500.13 MHz. The spectrometer was equipped with a 5-mm Bruker TCI Cryoprobe. Standard Bruker pulse sequences were used to record spectra in acetone-*d*_6_ at 300 K. Spectra were referenced to tetramethylsilane, which was used as an internal standard.

### 4.6. Analytical Data of 2-Methoxy-4-phenyl-1H-phenalen-1-one *(**5**)*

^1^H NMR (500 MHz, acetone-*d*_6_): δ 8.65 (1H, dd, *J* = 7.1, 1.2 Hz, H-9), 8.42 (1H, dd, *J* = 7.9, 1.2 Hz, H-7), 8.12 (1H, d, *J* = 8.5 Hz, H-6), 7.89 (1H, dd, *J* = 7.9, 7.1 Hz, H-8), 7.64 (1H, d, *J* = 8.5 Hz, H-5), 7.61–7.59 (5H, m, H-2’–H-6’), 7.10 (1H, s, H-3), 3.72 (3H, s, OMe). ^13^C NMR (125 MHz, acetone-*d*_6_, chemical shifts extracted from HMQC and HMBC): 179.0 (C-1), 153.8 (C-2), 142.7 (C-4), 140.4 (C-1’), 135.9 (C-7), 132.2 (C-6a), 130.8 (C-9), 129.1 (C-3’/C-5’), 130.2 (C-5), 130.2 (C-9a), 129.8 (C-6), 128.6 (C-4’), 130.7 (C-2’/C-6’), 127.3 (C-8), 125.5 (C-9b), 125.1 (C-3a), 111.1 (C-3), 55.2 (OMe). *HRESIMS*: *m*/*z* 287.1068 [M + 1]^+^ (calc. for C_20_H_15_O_2_ 287.1072).
